# Critical physiological factors influencing the outcome of antimicrobial testing according to ISO 22196 / JIS Z 2801

**DOI:** 10.1371/journal.pone.0194339

**Published:** 2018-03-20

**Authors:** Cornelia Wiegand, Andrea Völpel, Andrea Ewald, Markko Remesch, Jan Kuever, Janine Bauer, Stefanie Griesheim, Carolin Hauser, Julian Thielmann, Silke Tonndorf-Martini, Bernd W. Sigusch, Jürgen Weisser, Ralf Wyrwa, Peter Elsner, Uta-Christina Hipler, Martin Roth, Carolin Dewald, Claudia Lüdecke-Beyer, Jörg Bossert

**Affiliations:** 1 Klinik für Hautkrankheiten, Universitätsklinikum Jena, Jena, Germany; 2 Poliklinik für Konservierende Zahnheilkunde und Parodontologie, Universitätsklinikum Jena, Jena, Germany; 3 Lehrstuhl für Funktionswerkstoffe der Medizin und Zahnheilkunde, Universitätsklinikum Würzburg, Würzburg, Germany; 4 Amtliche Materialprüfungsanstalt (MPA), Abteilung Mikrobiologie, Bremen, Germany; 5 Thüringisches Institut für Textil- und Kunststoff-Forschung e.V., Rudolstadt, Germany; 6 Fraunhofer-Institut für Verfahrenstechnik und Verpackung IVV, Freising, Germany; 7 INNOVENT e.V., Bereich Biomaterialien, Jena, Germany; 8 Leibniz Institute for Natural Product Research and Infection Biology, Hans Knöll Institute, Bio Pilot Plant, Jena, Germany; 9 Lehrstuhl für Materialwissenschaft, Otto-Schott-Institut für Materialforschung, Jena, Germany; Natural Environment Research Council, UNITED KINGDOM

## Abstract

Bactericidal materials gained interest in the health care sector as they are capable of preventing material surfaces from microbial colonization and subsequent spread of infections. However, commercialization of antimicrobial materials requires proof of their efficacy, which is usually done using *in vitro* methods. The ISO 22196 standard (Japanese test method JIS Z 2801) is a method for measuring the antibacterial activity of daily goods. As it was found reliable for testing the biocidal activity of antimicrobially active materials and surface coatings most of the laboratories participating in this study used this protocol. Therefore, a round robin test for evaluating antimicrobially active biomaterials had to be established. To our knowledge, this is the first report on inaugurating a round robin test for the ISO 22196 / JIS Z 2801. The first round of testing showed that analyses in the different laboratories yielded different results, especially for materials with intermediate antibacterial effects distinctly different efficacies were noted. Scrutinizing the protocols used by the different participants and identifying the factors influencing the test outcomes the approach was unified. Four critical factors influencing the outcome of antibacterial testing were identified in a series of experiments: (1) incubation time, (2) bacteria starting concentration, (3) physiological state of bacteria (stationary or exponential phase of growth), and (4) nutrient concentration. To our knowledge, this is the first time these parameters have been analyzed for their effect on the outcome of testing according to ISO 22196 / JIS Z 2801. In conclusion, to enable assessment of the results obtained it is necessary to evaluate these single parameters in the test protocol carefully. Furthermore, uniform and robust definitions of the terms antibacterial efficacy / activity, bacteriostatic effects, and bactericidal action need to be agreed upon to simplify communication of results and also regulate expectations regarding antimicrobial tests, outcomes, and materials.

## Introduction

Bio-contamination of surfaces is one of the main vectors of infectious agents in the community and hospital sector. Pathogens are able to survive on surfaces for prolonged periods of time depending on the organism and the environmental conditions. They can be transferred easily to other objects in the environment by anyone or anything contacting these contaminated surfaces [1]. Thereby, scientific research on materials with intrinsic antimicrobial activity or antimicrobially active coatings was stimulated. Commercialization of antimicrobial materials and/or active coatings requires the proof of their effectivity. Hence, publications on material or coating developments are accompanied by reports on antimicrobial testing. These in vitro tests should allow a direct comparison of the effects of the materials on the micro-organisms. Ideally, they are simple, rapid, reproducible, inexpensive, and enable handling of a range of sample quantities [2]. However, the various test methods differ significantly in their properties and hence in their outcome. The results obtained are influenced by the method selected and the microorganisms used as well as by the extraction method and the degree of solubility or diffusibility of each test-compound [2], accordingly, accounting for inconsistencies in the reports on the antibacterial activity of biomaterials. Hence, it is difficult to compare the antimicrobial performance of different materials based on results of diverse study designs. Therefore, standard test methods have been developed to allow objective comparison of biomaterials and their antimicrobial surfaces. The ISO 22196 standard (Japanese test method JIS Z 2801) is a method for measuring the antibacterial activity of daily goods (International Organization for Standards 2007). It has been found reliable for testing the biocidal activity of antimicrobially active materials and surface coatings [3, 4] and most of the laboratories that participated in the present study use this protocol. Nonetheless, there are only few reports in the literature reporting upon either the ISO 22196 or the JIS Z 2801. A Pubmed search yielded 22 hits searching for ‘ISO 22196’ OR ‘JIS Z 2801’ with eleven articles for each. A critical review of these articles, however, revealed that most of the studies employed modified versions of these standards or did not give a detailed description of the protocol used. Table 1 summarizes the requirements for the JIS Z 2801 film covering method [5] and compares it to the information obtained from the publications reviewed.

**Table 1:** Comparison for the outline of JIS Z 2801:2000 / ISO 22196 [5] and the protocols used in the different studies.

item	study protocols given in the material and methods sections	
**bacteria**	used *E*. *coli* and *S*. *aureus*: 13	[3], [4], [6], [7], [8], [9], [10], [11], [12], [13], [14], [15], [16]
	other microorganisms included: 8	[17], [18], [19], [20], [21], [22], [23], [24]
	additional microorganisms employed: 6	[4], [6], [7], [9], [10], [11]
**preparatory incubation of bacteria**	no information: 20	[3], [4], [6], [7], [8], [9], [10], [11], [12], [13], [15], [16], [17], [18], [19], [20], [21], [22], [23], [24]
	inconsistent with standard: 1	[14]
**cell concentration of inoculum**	no information: 8	[3], [4], [6], [13], [15], [16], [20], [24]
	according to standard: 7	[8], [12], [14], [17], [18], [19], [21]
	higher than standard: 5	[7], [9], [10], [11], [22]
	lower than standard: 1	[23]
**solution for bacterial suspension**	no information: 16	[3], [4], [6], [7], [8], [13], [15], [16], [17], [18], [19], [20], [21], [22], [23], [24]
	according to standard: 4	[9], [10], [11], [12]
	lower dilution factor: 1	[14]
**specimen**	no information: 7	[3], [4], [8], [17], [19], [22]
	according to standard: 5	[6], [7], [9], [12], [13]
	other dimensions (smaller, different shape): 9	[10], [11], [14], [16], [18], [20], [21], [23], [24]
**incubation**inoculum	no information: 10	[3], [4], [8], [13], [14], [15], [17], [19], [22], [24]
	according to standard: 3	[6], [12], [21]
	other amounts (lower/larger): 7	[9], [10], [11], [16], [18], [20], [23]
	different application method: 1	[7]
temperature	no information: 6	[3], [4], [8], [13], [15], [16]
	according to standard: 5	[9], [12], [19], [21], [22]
	at 37°C: 8	[6], [7], [10], [11], [14], [17], [18], [20]
	at 25°C: 2	[23], [24]
time	no information: 4	[3], [4], [13], [15]
	according to standard: 16	[6], [8], [9], [10], [11], [12], [14], [16], [17], [18], [19], [20], [21], [22], [23], [24]
	others: 1	[7]
	additional time points: 3	[9], [10]

A round robin test for the evaluation of antimicrobially active biomaterials was therefore established. To our knowledge, this is the first report on establishing a round robin test for the ISO 22196 / JIS Z 2801. The following laboratories participated: (1) Klinik für Hautkrankheiten, Universitätsklinikum Jena, Germany, (2) Amtliche Materialprüfanstalt (MPA), Abteilung Mikrobiologie, Bremen, Germany, (3) Fraunhofer-Institut für Verfahrenstechnik und Verpackung IVV, Freising, Germany, (4) Thüringisches Institut für Textil- und Kunststoff-Forschung e.V. (TITK), Rudolstadt, Germany, (5) Poliklinik für Konservierende Zahnheilkunde und Parodontologie, Universitätsklinikum Jena, Germany, (6) Lehrstuhl für Materialwissenschaft, Otto-Schott-Institut für Materialforschung, Jena, Germany, (7) INNOVENT e.V., Bereich Biomaterialien, Jena, Germany, and (8) Lehrstuhl für Funktionswerkstoffe der Medizin und Zahnheilkunde, Universitätsklinikum Würzburg, Germany. The material to be tested was kindly provided by the TITK. Subsequently to the first round of testing it became clear that analyses in the different laboratories yielded different results. Not only did the different labs use diverse reporting systems, e.g. convey the decrease in bacterial numbers in log units or percent, but especially for a material with intermediate antibacterial effects distinctly different efficacies were noted. Therefore, we set out to unify the approach identifying the factors influencing test outcomes by evaluation of differences in protocols concerning medium, cell number, time and microorganisms used. To our knowledge, this is the first time these parameters have been systematically analyzed for their effect on the outcome of testing according to ISO 22196 / JIS Z 2801.

## Materials and methods

### Materials

Reference (without antibacterial activity) and test compounds (with presumed antibacterial activity) were provided by Thüringisches Institut für Textil- und Kunststoff-Forschung e.V.. The compounds 1, 2 and 3 consisted of polyamide 6 (PA6) and the antibacterial zinc additive developed and patented by TITK (patent: EP2140958) in various concentrations (compound 1 = 0.5% zinc additive; 2 = 2.5% zinc additive; 3 = 5% zinc additive). Compounds were produced as follows: First a master batch was fabricated by extrusion. Afterwards, test specimens (plates) were manufactured by injection molding utilizing a certain amount of the master batch. Reference was produced similarly without supplementation with the zinc additive. The materials were dried at 80°C in a vacuum oven before the respective processing stages. Immediately after the injection molding, the samples were welded in aluminum foil remaining in this packaging until the start of the test. This was necessary to prevent water absorption and degradation phenomena. In this way, a uniform sample quality could be ensured in spite of different round robin test start times at the individual participating laboratories.

All participating laboratories received the materials not knowing the composition. Furthermore, all laboratories used the test microbe *E*. *coli* DSM 498 (provided by Leibniz Institute for natural Product Research and Infection Biology, Jena, Germany).

### Characterisation of the materials surfaces

To characterize the topography of the materials surfaces atomic force microscopy (AFM) in tapping mode was used. All measurements were performed in air with a Dimension 3100 (Bruker, Santa Barbara, CA, USA) equipped with a Nanoscope IV controller. Silicon cantilever Bruker RTESP (Bruker, Santa Barbara, CA, USA) with a resonance frequency of 315–364 kHz, a spring constant between 20–80 N/m and a maximal tip radius of 12 nm was used. The scan size was 2 μm × 2 μm and the scan rate was 2 Hz at three randomly chosen positions of each sample. For image analysis, the NanoScope Analysis software 1.5 (Bruker, Santa Barbara, CA, USA) was used.

The contact angles were obtained by dynamic sessile drop method using a drop shape analysis system (DSA 10Mk2 Drop shape analysis system, Krüss, Hamburg, Germany). Deionized water with a flow rate of 10 μL/min and a drop volume of 2.5 μL were used to perform measurements on three randomly chosen positions of each sample. Ten images per run were recorded by a camera (n = 30 images per each sample) and analyzed according to the tangent method using software supplied by the manufacturer.

#### Testing of antibacterial activity

Performance testing of the compounds was principally conducted in accordance with ISO 22196:2011 and JIS Z 2801:2012. The ISO 22196:2011 stipulates the procedure for testing of antibacterial activity of plastics and states the ranges of test parameters. It determines the preparation of test specimens, culture conditions for test microbes, the incubation time, the content of nutrient medium, and the analytical method. However, the standards are not freely accessible and need to be bought. To avoid any legal repercussions we refer to the standard, all changes to the standard protocol are given in detail to clarify the experimental procedures. Table 2 denotes respective deviations regarding media and workflow procedures. In addition, some of the laboratories carried out alternative test methods which are described briefly in Table 3.

**Table 2:** General information on test conditions according to ISO 22196:2011/ JIS Z 2801:2012 and deviations from the standard protocol.

Laboratory	1	2	3	4	5	6	7	8
Sample preparation	according to standard	according to standard	according to standard	according to standard	according to standard	other dimensions: 15 mm x 15mm	according to standard	other dimensions: round 15 mm
Microorganism	*Escherichia coli* (DSM 498)	*Escherichia coli* (DSM 498)	*Escherichia coli* (DSM 498)	*Escherichia coli* (DSM 498)	*Escherichia coli* (DSM 498) according to standard	*Escherichia coli* (DSM 498)	*Escherichia coli* (DSM 498)	*Escherichia coli* (DSM 498)
Medium	Columbia agar, Caso- broth	TS—agar	TS–agar, TS—broth	according to standard	according to standard	M9 –mineral broth	Soya bean Casein Digest Medium acc. to EP/USP	LB—broth
cell count inoculum	lower than standard 5–7 x 10^4^	higher than standard	according to standard	according to standard	according to standard	OD_600_ = 0.5	according to standard	according to standard
Suspension medium	NB 1/20	according to standard	according to standard	according to standard	according to standard	0.85% NaCl	according to standard	according to standard
Number of replicates	according to standard	according to standard	according to standard	according to standard	according to standard	3	2 biological replicates and 2 technical replicates each	6
Incubation (temperature/time)	according to standard	according to standard	according to standard	according to standard	according to standard	24 h/28°C	according to standard	according to standard
Extraction medium	0.9% NaCl with 0.2% Tween	NB, 0.01% TWEEN 80	¼ Ringer’s solution 0,2% Tween	according to standard	PBS	NaCl	PBS	
Determination	CFU	CFU	CFU	CFU	CFU	CFU and live/dead staining	CFU	CFU

NB–Nutrient Broth; CFU–Colony Forming Unit

**Table 3:** Description of alternative test methods used in the study.

Laboratory	1	6	7	8
Test	extraction-based MLN test Microplate laser nephelometry	live / dead assay	BacTiter-Glo (BTG) test (Promega) combined with CFU assay	WST 1 test (Roche Diagnostics, Mannheim, Germany)
Experimental design	incubate bacterial suspensions with extracts of test materials (0.2 g / mL)	cultivated bacteria (OD_600_ 0.5) in direct contact with test materials	Incubation of bacterial suspension in contact with the test materials by help of flexiPERM silicone masks	Inoculum 1x10^4^ / 1x 10^6^, ONC / log phase, NB 1/500 / 1/200, cultivated bacteria in direct contact with test materials
determination	scattered light intensity of colloidal suspension → determination of bacterial proliferation	fluorescence intensity in CLSM	BTG: quantitation of bacterial ATP → CFU according to common method	Reduction of WST-1 reagent by cellular dehydrogenases →bacterial activity
outcome	release of antimicrobial components	viability of adherent bacteria on material surface and supernatant	CFU: different increase in cell number, BTG: different levels of ATP	percentage of surviving bacteria on the sample surface

## Results and discussion

### Start of the round robin tests

Subsequent to the first round of testing it became clear that outcomes differed between the laboratories participating in the round robin test (Table 4). Although seven out of eight laboratories chose testing according to ISO 22196 / JIS Z 2801, the results varied, especially for the material with intermediate effects. In addition, four alternative tests were performed by the participating laboratories. This also resulted in disparities in the reporting systems. Only laboratories 2, 3, and 4 stated antimicrobial efficacy in R values while the other laboratories reported bacterial decrease in log-reduction or percent inhibition as well as live/dead ratios. However, the alternative tests pointed out interesting characteristics of the material tested. From the extraction-based MLN test (laboratory 1), we learned that the zinc additive is not released from the compounds. Hence, antibacterial activity will only ensue if the bacteria come into close contact with the material. Yet, challenging with high amounts of bacteria resulted in a low antibacterial activity leading to possible overgrowth of the material (laboratory 6). In addition, only a bacteriostatic effect was detectable in the experiments in which flexiPERM silicone masks were used to ensure a defined area of exposure and in which ATP levels were measured in parallel from aliquot parts besides CFU values (laboratory 7). Starting with a cell density of 0.6 x 10^6^ bacteria/mL after 24 h of incubation the cell densities rose to more than 10 x 10^6^ bacteria/mL at the negative control and at compound 1 which was not determined more exactly since an inhibition was expected. Only at compound 3 a value of 8.4 x 10^6^ bacteria/mL could be counted and calculated for the bacterial density in the flexiPERM chambers. Under these conditions the measurement of ATP allowed a better quantification. The interpretation of reductions of the ATP level depends on the general conditions, under experimental conditions characterized by a stagnation of the cell numbers, e. g. by nutritional or temporal limitations, a reduction of the measured total ATP in comparison to the nearly constant control indicates a lowered intracellular ATP level. In metabolically injured cells it is caused by reduction of the ATP synthesis at still occurring consumption of ATP. Also a decay of ATP is possible by ATPases and non-specific chemical processes. In these cases, the reduction of ATP is a clear sign of injury of cells [25]. On the other hand, also in damaged or even dead cells degradation and decay of ATP could be slowed down maintaining residual ATP despite of damage. Therefore, it is often difficult to correlate the percentage of remaining ATP with a defined log-reduction in the CFU values. This is only possible in case of a well-defined bactericidal activity [25]. Nevertheless, even a minimal impairment of the ATP level is an indicator of cell damaging influences or processes in most cases. An entirely different situation exists if cells were still proliferating and cell number was increasing with time. Then, differences of measured total ATP are based on different cell numbers. Proliferative cells have to maintain an averaging intracellular ATP level, notwithstanding that a larger diversity of the intracellular ATP between individual bacterial cells has been found [26]. Especially in case of unknown or insufficiently characterized antibacterial activities, measurement of ATP should be correlated with the estimation of CFU values.

**Table 4:** Summary of the results for antibacterial testing of the compounds in different laboratories.

laboratory	compound	result	assessment
**1**	**1**	contact test: log-reduction = 2	++
		extraction-based test: 0% inhibition	no effect
	**2**	contact test: log-reduction = 5	+++
		extraction-based test: 0% inhibition	no effect
	**3**	contact test: log-reduction = 6	+++
		extraction-based test: 0% inhibition	no effect
**2**	**1**	0.49 R	no effect
	**2**	5.58 R	+++
	**3**	5.58 R	+++
**3**	**1**	5.66 R	+++
	**2**	n.d.	
	**3**	5.66 R	+++
**4**	**1**	0.5 R	+
	**2**	1.73 R	+
	**3**	5.62 R	+++
**5**	**1**	n.d.	
	**2**	log-reduction = 6.3	+++
	**3**	log-reduction = 6.3	+++
**6**	**1**	n.d.	
	**2**	1.1% dead	+
	**3**	6.1% dead	+
**7**	**1**	40% living	++
	**2**	n.d.	
	**3**	10% living	+++
**8**	**1**	0% inhibition	no effect
	**2**	60% inhibition (LB)	+
		100% inhibition (LB 1:500)	+++
	**3**	45% inhibition (LB)	++
		100% inhibition (LB 1:500)	+++

+ slight antibacterial effect

++ significant antibacterial effect

+++ strong antibacterial effect

n.d. not determined

### Factors influencing antimicrobial testing outcome

**Fig 1:**
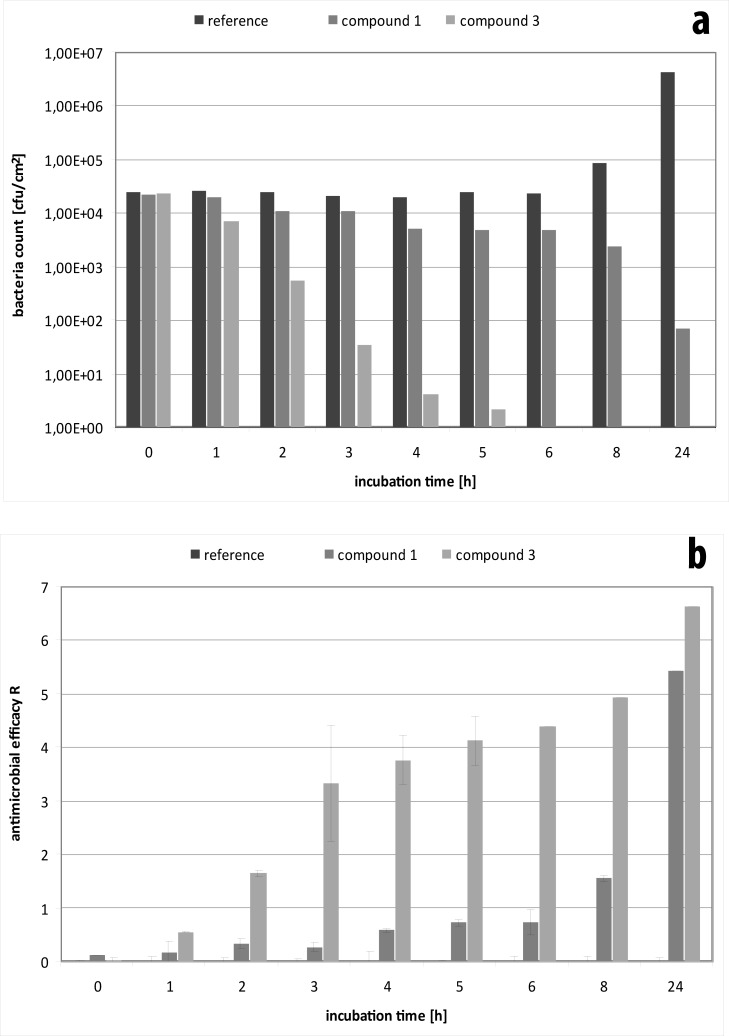
Testing of the effect of compound 1 and 3 on *E*. *coli* subject to incubation time (a) and calculation of the resulting R values according to ISO 22196 / JIS Z 2801 (b). Data is given as mean ± standard deviation. Note that the R values were calculated to the growth controls at the respective time points to avoid bias.

Discussion among the round robin test members led to the identification of possible influence factors from the differences in the execution of the tests such as medium dilution, bacteria number and physiological state of the microorganisms used as well as the time. The standard incubation time for the test is twenty-four hours. It was found that antibacterial efficacy exhibits a distinct dependency on the incubation time (Fig 1). The effect is most pronounced for the compound 1, which demonstrated a significant increase in impact from eight to twenty-four hours. Compound 3 also showed an enhancement of activity against *E*. *coli* over time resulting in complete eradication of bacteria after six hours.

**Fig 2:**
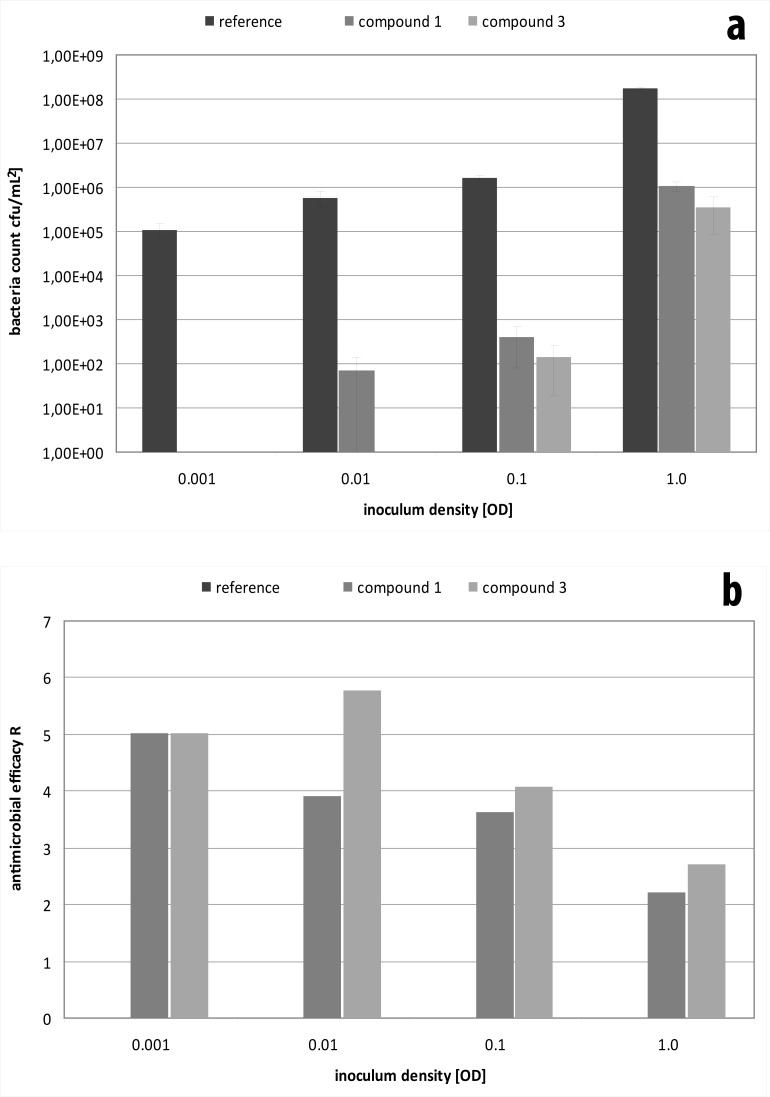
Determination of the antibacterial effect of compound 1 and 3 on *E*. *coli* after twenty-four hours as a function of the inoculum density by measurement of the bacterial count (a) and calculation of the resulting R values according to ISO 22196 / JIS Z 2801 (b). Data is given as mean ± standard deviation.

Moreover, starting concentrations of bacteria affected the outcome of the test significantly (Fig 2). Low inoculum amounts resulted in a high antibacterial efficacy of both compounds 1 and 3. Increasing bacterial concentrations from 10^4^/cm^2^ to 10^5^/cm^2^ led to a loss of the bactericidal effect of compound 1. Further increment yielded surviving bacteria on compound 3, too, and at 10^7^/cm^2^ both compounds only exhibited an antibacterial effect of less than a three log-reduction.

**Fig 3:**
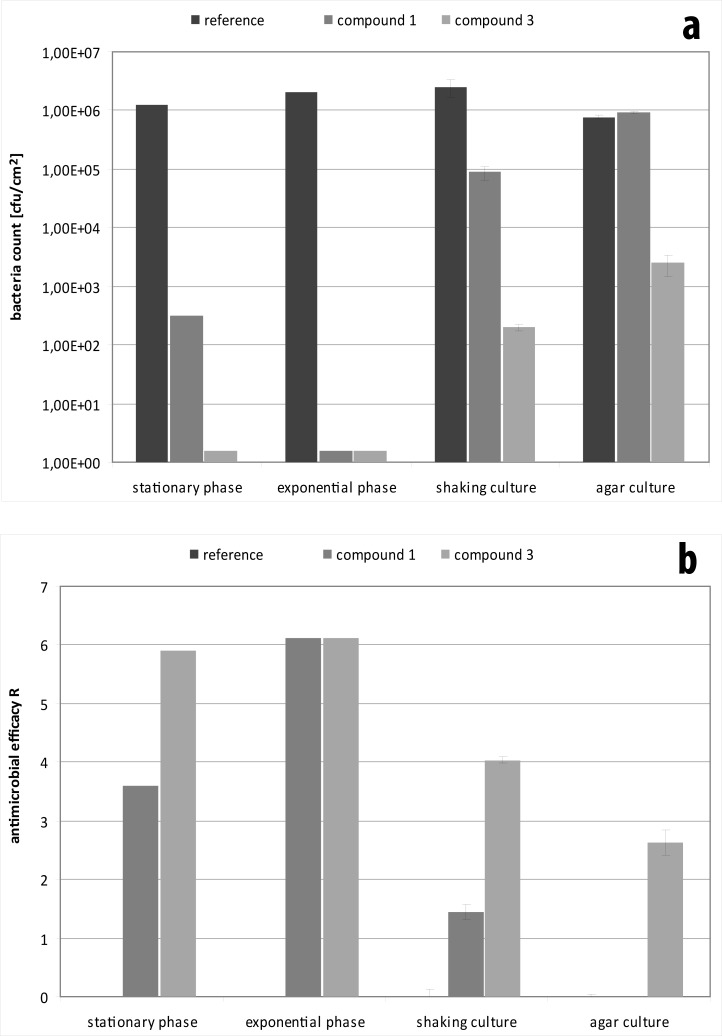
Assessment of the physiological state of the microorganisms on the outcome of antibacterial testing of compound 1 and 3. Therefore, *E*. *coli* in the stationary phase and the exponential growth phase were compared as well as *E*. *coli* grown in shaking cultures over night or colonies used directly from agar plates. *E*. *coli* was monitored over twenty-four hours (a) and resulting R values were calculated after twenty-four hours according to ISO 22196 / JIS Z 2801 (b). Data is given as mean ± standard deviation.

In addition, it plays a distinct role if the bacteria are in the stationary or exponential phase of growth (Fig 3). This was confirmed in tests for materials with intermediate activity like compound 1. Against stationary bacteria compound 1 was less effective (R = 3.61) compared to compound 3 (R = 5.91). In contrast, both exhibited a log-reduction greater six when used against bacteria in the exponential phase. This is also the case when the inoculum is prepared directly from colonies grown on agar plates compared to those grown in an overnight shaking culture (Fig 3).

**Fig 4:**
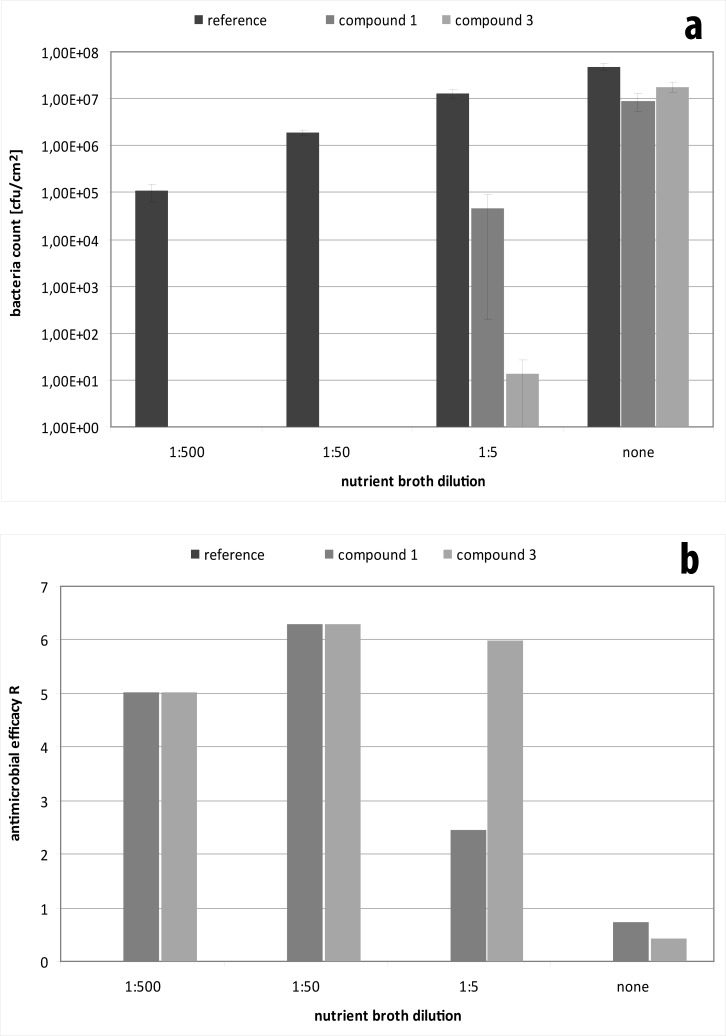
Evaluation of the influence of compound 1 and 3 on *E*. *coli* after twenty-four hours as a function of nutrient supply. Experiments were performed in nutrient broth diluted 1:500, 1:50, and 1:5 compared to undiluted and resulting amounts of *E*. *coli* were measured (a) and R values calculated according to ISO 22196 (b). Data is given as mean ± standard deviation.

In a similar way, availability of nutrients affects the test outcome. While the standard test uses a high dilution of the nutrient broth for incubation to avoid exuberant growth of bacteria, the use of higher amounts of nutrients resulted in a pronounced growth of bacteria (Fig 4). Vice versa, antibacterial activity of the materials declined from being greater than a three log-reduction to less than one log-reduction with increasing nutrient concentration.

### Characterization of the material surfaces

**Fig 5:**
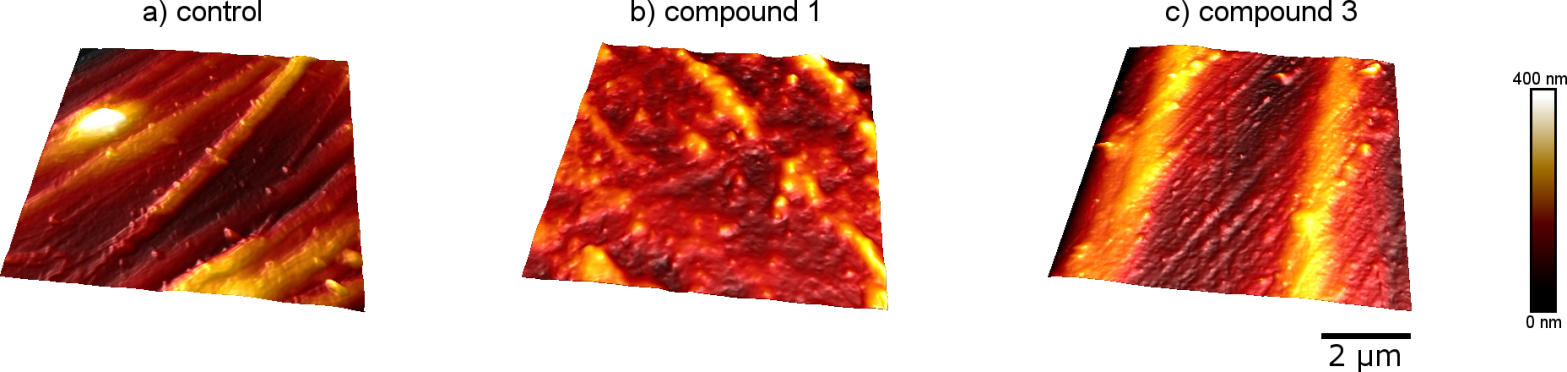
AFM measurements of the PA6 control (a), compound 1 (b) and compound 3 (c). Surface roughness is depicted in a range from 0 to 400 nm. The scale bar determines a length of 2 μm.

For determination of the surface topography, the material surfaces were characterized with an AFM (Fig 5). The roughness of compound 1 was R_q_ = 58 ± 10 nm and for compound 3 R_q_ = 101 ± 11 nm (Table 5). The control samples had a roughness of R_q_ = 56 ± 9 nm. The results indicate that the compound 1 and the control sample had a similar roughness, whereas, the compound 3 specimen showed a higher roughness value. Wettability of the investigated materials was characterized by contact angle measurements. The contact angle was determined to be 79 ± 5° for compound 1 and 78 ± 62° for compound 3 while the PA6 control sample featured an angle of 89 ± 2°. Based on the contact angle measurements, compound 1 and 3 were slightly hydrophilic, whereas, the control sample was more hydrophobic.

**Table 5:** Summary of material surface characterization for the compounds.

	PA6 control	compound 1	compound 3
**surface roughness (R**_**q**_**) [nm]**	56 ± 9	58 ± 10	101 ± 11
**contact angle [°]**	89 ± 2	79 ± 5	78 ± 6

Material surface characterization showed that the augmentation with the zinc additive changed surface properties compared to the PA 6 control sample. The compounds exhibited higher roughness values and were found to be more hydrophilic. Roughness seemed to increase with the amount of the zinc additive. It has been previously observed that surfaces from transition metal oxides such as ZnO demonstrate an increase in antimicrobial activity with higher roughness [27]. This could also be shown for antifungal efficacy of titanium discs with amphotericin B at different surface roughness [28] as well as for the bactericidal effects of silver dopings [29]. Hence, it could be concluded that with increasing surface roughness release kinetics improve. It would be of interest to further investigate the materials tested with regard to this feature.

## Conclusions

Application of antimicrobial materials and/or active coatings requires the proof of their effectivity. The ISO 22196 standard (Japanese test method JIS Z 2801) is a method for measuring the antibacterial activity of daily goods (International Organization for Standards 2007). It has been found reliable for testing the biocidal activity of antimicrobially active materials and surface coatings [3, 4] and most of the laboratories that participated use this protocol. The incentive of the present study was the establishment of a round robin test for the evaluation of antimicrobially active biomaterials using the ISO 22196 / JIS Z 2801. To our knowledge, this is the first report on such an endeavor. It could be shown that a high zinc additive amount in the test compounds exhibits a distinct antibacterial effect. Yet, the first series of the round robin test likewise showed that results varied considerably although seven out of eight laboratories chose testing according to ISO 22196 / JIS Z 2801. Four critical factors influencing the outcome of antibacterial testing could be identified in the series of experiments: (1) incubation time, (2) starting concentration of the bacteria as well as (3) physiological state of the bacteria (stationary or exponential phase of growth), and (4) concentration of the nutrients. It became apparent that all changes in the protocol need to be listed meticulously to enable evaluation of the results obtained. It could be considered to advocate the restriction of the tolerances in the DIN norm to ensure the collection of serious data. In addition, discussions on the test outcomes revealed that there are no clear definitions on the terms antibacterial efficacy / activity, bacteriostatic effects, and bactericidal action. This could be observed in the various descriptions of the results ranging from stating the R value or log-reduction to the allegation of inhibition in [%] and the amount of living or dead bacteria given in [%] and their subsequent different assessment. Uniform and robust definitions should be agreed upon and defined in the ISO 22196 / JIS Z 2801 standard to simplify not only communication of results but also regulate expectations concerning antimicrobial tests, outcomes, and materials.

## Supporting information

S1 Table Data for testing of compound 1 and 3 against *E*. *coli* subject to incubation time. (DOCX)

S2 Table Data for testing of compound 1 and 3 against *E*. *coli* after twenty-four hours as a function of the inoculum density. (DOCX)

S3 Table Data for testing of compound 1and 3 against *E*. *coli* in the stationary phase and the exponential growth phase as well as *E*. *coli* grown in shaking cultures over night or colonies used directly from agar plates. (DOCX)

S4 Table Data for testing of compound 1 and 3 againt *E*. *coli* as a function of nutrient supply using nutrient broth diluted 1:500, 1:50, and 1:5 compared to undiluted nutrient broth. (DOCX)

## Abbreviations

AFM: atomic force microscopy
ISO: International Organization for Standardization
JIS: Japanese Industrial Standard
