# Supplementary material for: Critical physiological factors influencing the outcome of antimicrobial testing according to ISO 22196 / JIS Z 2801
Source: PLoS One. 2018 Mar 20;13(3):e0194339. doi: 10.1371/journal.pone.0194339 (PMC5860763; doi:10.1371/journal.pone.0194339)
Supplement: S1 Table — (DOCX) [file pone.0194339.s001.docx]

S1 Table. Data for testing of compound 1 and 3 against *E. coli* subject to incubation time.

|  | incubation time [h] | 0 | 1 | 2 | 3 | 4 | 5 | 6 | 8 | 24 |
| --- | --- | --- | --- | --- | --- | --- | --- | --- | --- | --- |
| growth control | cfu/cm^2^ | 2.40E+04 | 2.50E+04 | 2.40E+04 | 2.10E+04 | 2.00E+04 | 2.40E+04 | 2.30E+04 | 8.40E+04 | 4.30E+06 |
| compound 1 | cfu/cm^2^ | 2.20E+04 | 1.90E+04 | 1.10E+04 | 1.10E+04 | 5.10E+03 | 4.70E+03 | 4.80E+03 | 2.30E+03 | 6.90E+01 |
| compound 3 | cfu/cm^2^ | 2.30E+04 | 7.00E+03 | 5.30E+02 | 3.40E+01 | 4.20E+00 | 2.10E+00 | 0.00E+00 | 0.00E+00 | 0.00E+00 |
| growth control | R | 0 | 0 | 0 | 0 | 0 | 0 | 0 | 0 | 0 |
| compound 1 | R | 0.12 | 0.16 | 0.33 | 0.27 | 0.58 | 0.72 | 0.73 | 1.55 | 5.41 |
| compound 3 | R | 0.02 | 0.55 | 1.64 | 3.32 | 3.75 | 4.12 | 4.38 | 4.92 | 6.63 |
