# Supplementary material for: Critical physiological factors influencing the outcome of antimicrobial testing according to ISO 22196 / JIS Z 2801
Source: PLoS One. 2018 Mar 20;13(3):e0194339. doi: 10.1371/journal.pone.0194339 (PMC5860763; doi:10.1371/journal.pone.0194339)
Supplement: S2 Table — (DOCX) [file pone.0194339.s002.docx]

S2 Table. Data for testing of compound 1 and 3 against *E. coli* after twenty-four hours as a function of the inoculum density.

|  | inoculum densitiy [OD] | 0.001 | 0.01 | 0.1 | 1.0 |
| --- | --- | --- | --- | --- | --- |
| growth control 0h | | 1.69E+04 | 1.42E+05 | 3.14E+05 | 1.40E+07 |
| growth control | cfu/cm^2^ | 1.06E+05 | 5.84E+05 | 1.69E+06 | 1.75E+08 |
| compound 1 | cfu/cm^2^ | 0.00E+00 | 7.22E+01 | 4.00E+02 | 1.07E+06 |
| compound 3 | cfu/cm^2^ | 0.00E+00 | 0.00E+00 | 1.41E+02 | 3.48E+05 |
| growth control | R | 0.00 | 0.00 | 0.00 | 0.00 |
| compound 1 | R | 5.03 | 3.91 | 3.62 | 2.21 |
| compound 3 | R | 5.03 | 5.77 | 4.08 | 2.70 |
