# Supplementary material for: Critical physiological factors influencing the outcome of antimicrobial testing according to ISO 22196 / JIS Z 2801
Source: PLoS One. 2018 Mar 20;13(3):e0194339. doi: 10.1371/journal.pone.0194339 (PMC5860763; doi:10.1371/journal.pone.0194339)
Supplement: S3 Table — (DOCX) [file pone.0194339.s003.docx]

S3 Table. Data for testing of compound 1and 3 against *E. coli* in the stationary phase and the exponential growth phase as well as *E. coli* grown in shaking cultures over night or colonies used directly from agar plates.

|  |  | stationary phase | exponential phase | shaking culture | agar culture |
| --- | --- | --- | --- | --- | --- |
|  |  |  |  |  |  |
| growth control | cfu/cm^2^ | 1,26E+06 | 2,02E+06 | 2,51E+06 | 7,75E+05 |
| compound 1 | cfu/cm^2^ | 3,13E+02 | 1,56E+00 | 8,79E+04 | 9,31E+05 |
| compound 3 | cfu/cm^2^ | 1,56E+00 | 1,56E+00 | 2,00E+02 | 2,49E+03 |
| growth control | R | 0,00 | 0,00 | 0,00 | 0,00 |
| compound 1 | R | 3,61 | 6,11 | 1,44 | -0,08 |
| compound 3 | R | 5,91 | 6,11 | 4,04 | 2,63 |
