# Supplementary material for: Critical physiological factors influencing the outcome of antimicrobial testing according to ISO 22196 / JIS Z 2801
Source: PLoS One. 2018 Mar 20;13(3):e0194339. doi: 10.1371/journal.pone.0194339 (PMC5860763; doi:10.1371/journal.pone.0194339)
Supplement: S4 Table — (DOCX) [file pone.0194339.s004.docx]

S4 Table. Data for testing of compound 1 and 3 againt *E. coli* as a function of nutrient supply using nutrient broth diluted 1:500, 1:50, and 1:5 compared to undiluted nutrient broth.

|  | nutrient broth dilution | 1:500 | 1:50 | 1:5 | none |
| --- | --- | --- | --- | --- | --- |
| growth control 0h | | 1,69E+04 | 2,30E+04 | 1,97E+04 | 2,80E+04 |
| growth control | cfu/cm^2^ | 1,06E+05 | 1,93E+06 | 1,28E+07 | 4,79E+07 |
| compound 1 | cfu/cm^2^ | 0,00E+00 | 0,00E+00 | 4,64E+04 | 8,99E+06 |
| compound 3 | cfu/cm^2^ | 0,00E+00 | 0,00E+00 | 1,31E+01 | 1,80E+07 |
| growth control | R | 0,00 | 0,00 | 0,00 | 0,00 |
| compound 1 | R | 5,03 | 6,28 | 2,44 | 0,73 |
| compound 3 | R | 5,03 | 6,28 | 5,99 | 0,42 |
